# Hypoxia-Inducible Factor 1α and 2α Have Beneficial Effects in Remote Ischemic Preconditioning Against Stroke by Modulating Inflammatory Responses in Aged Rats

**DOI:** 10.3389/fnagi.2020.00054

**Published:** 2020-03-10

**Authors:** Xiangnan Du, Jian Yang, Cuiying Liu, Sainan Wang, Chencheng Zhang, Heng Zhao, Huishan Du, Xiaokun Geng

**Affiliations:** ^1^Department of Neurology, Beijing Luhe Hospital, Capital Medical University, Beijing, China; ^2^China-America Institute of Neuroscience, Department of Neurology, Beijing Luhe Hospital, Capital Medical University, Beijing, China; ^3^Department of Neurosurgery, School of Medicine, Stanford University, Stanford, CA, United States

**Keywords:** cerebral ischemia, remote ischemic preconditioning, hypoxia-inducible factor, Akt signaling pathway, inflammatory cytokines

## Abstract

Limb remote ischemic preconditioning (RIPC) has been proven to alleviate stroke injury in young rats, but its protective effect and its mechanism in aged rats are still unclear. Hypoxia-inducible factor (HIF) is one of the important markers of stroke, and its high expression plays an important role in the pathogenesis of stroke. In this study, we tested the hypothesis that RIPC could regulate the expression of HIF, leading to reduced inflammatory responses in aged rats. Stroke was induced by transient middle cerebral artery occlusion (MCAo) in aged rats, and RIPC was conducted in both hind limbs. The HIF-1α and HIF-2α mRNA and protein were examined by real-time RT-PCR and western blotting (WB). Inflammatory cytokines in the peripheral blood and brain were measured using AimPlex multiplex immunoassays. The protein levels of p-Akt, Akt, p-ERK, and ERK were examined by WB. We investigated that RIPC reduced the infarct size, improved neurological functions, and decreased the expression of HIF-1α and HIF-2α in the ischemic brain. RIPC reduced the levels of IL-1β, IL-6 and IFN-γ in the peripheral blood and the levels of IL-1β and IFN-γ in the ischemic brain 48 h post-stroke. Moreover, intraperitoneal injection of the HIF inhibitor, acriflavine hydrochloride (ACF), abolished the protection of RIPC with respect to infarct size and neurological functions and neutralized the downregulation of pro-inflammatory IL-1β, IL-6 and IFN-γ. ACF also reversed the activation of the Akt signaling pathway induced by RIPC following stroke. HIF may play a key role in RIPC, which was likely mediated by the Akt signaling pathway and systemic modulation of the inflammatory response in aged rats.

## Introduction

Ischemic stroke is considered to be the third most lethal and disabling disease in the world. Aging, above all risk factors of stroke, accounts for a large proportion of stroke-related mortality (Mozaffarian et al., 2015). According to the World Health Organization (WHO) statistics, twelve million people will suffer from stroke in the coming decade (Krishnamurthi et al., 2013). At present, however, no alternative neuroprotective agent has been approved by the FDA except for thrombolytic recombinant tissue plasminogen activator (tPA), which is only administrated to a small number of patients. Given the increase of the senior citizen and the aggravating of the associated stroke burden, it is urgent to find effective treatments for this disease. In recent years, remote ischemic preconditioning (RIPC) has become a feasible therapeutic strategy for stroke and has been proven to provide a protective effect on young experimental animals (Wei et al., 2012; Liu et al., 2016; Yang et al., 2018) and in clinical trials (Mi et al., 2016; Zhao S. C. et al., 2017). Nevertheless, its protective effect and its mechanism in the elderlies are still unclear.

Age is an important risk factor for pre-clinical studies on ischemic stroke, which has been largely overlooked. Previous studies have suggested that aged rats, compared with younger ones, represent a more clinically relevant model of ischemic stroke (Mayhan et al., 2008; Modrick et al., 2009). Advanced age increases the expression of immune cells including T cells, neutrophils, macrophages, and dendritic cells in middle cerebral artery occlusion (MCAo) mice, which may be responsible for the exacerbated behavioral deficits in the aged (Manwani et al., 2013). Age also stimulates the activation of macrophages and the expression of inflammatory cytokines in MCAo mice (Zhao S. C. et al., 2017). Although most experimental stroke studies have been conducted on healthy young animals, accumulating evidence points to age-related differences in gene expression and cytokine production (Kuzumaki et al., 2010; Semple et al., 2013; Orre et al., 2014; Suenaga et al., 2015); thus, applying aged animals as models may increase the clinical relevance of the experimental results.

Hypoxia-inducible factor (HIF) is the most crucial regulator of oxygen balance in mammals, and it has been shown to promote tolerance to acute ischemia (Semenza, 2012). HIF-1α and HIF-2α, as the HIF-α subunits, combine with HIF-β to regulate target gene transcription under hypoxic conditions. They share 48% identical DNA binding regions in structure, but each has its own unique biological effects. HIF can easily degrade under normal oxygen conditions within 5 min, while under hypoxia, its stability and transcriptional activity are significantly increased, which induces the expression of its downstream target genes. Because of its particularity of function, HIF is critically related to ischemic stroke (Sharp and Bernaudin, 2004; Ogle et al., 2012). Previous studies showed that suppressing the expression of basal HIF-2α, but not that of HIF-1α, was sufficient to accelerate cell death in nerve growth factor (NGF)-stimulated neurons (Lomb et al., 2009). In addition, the HIF-α subunits HIF-1α, HIF-2α and HIF-3α can be regulated by the phosphoinositide 3-kinase (PI3K) pathway in the brain of MCAo rats (Wei et al., 2017). The interaction between RIPC and HIF has been explored by some studies, where HIF was required for RIPC in protecting heart function in young animal models (Cai et al., 2013; Martin-Puig et al., 2015). Our previous study showed that HIF-1α was indispensable for RIPC against stroke by modulating inflammation in a young rat model of MCAo. Accumulating data provide indications for the roles of HIF-α in neuroprotection of RIPC against ischemia, but there is still a lack of solid evidence of its involvement in an aged model of stroke with RIPC.

After a stroke, systemic inflammation contributes to brain injury, as neuroinflammation occurs not only in the brain but also in the peripheral organs (Nguyen et al., 2016; Shim and Wong, 2016; Yamashiro et al., 2017). RIPC could reduce neuroinflammation in ischemic stroke through modulating the upregulated expression of HIF-1α (Yang et al., 2018), improving the peripheral immune cell response (Liu et al., 2016), activating the adenosine A1 receptor and affecting redox status (Hu et al., 2012), and changing the activation of signaling pathways such as Akt and ERK. All of these findings have focused on young animal models, whereas whether RIPC can regulate neuroinflammation in aged animals remains unclear.

In this study, we aimed to provide insights into the roles of HIF-1α and HIF-2α in RIPC protection in aged stroke rats and explore the regulatory mechanism of inflammatory cytokines. First, we defined the protection of RIPC on aged MCAo rats. Then, we tested the expression of HIF-1α and HIF-2α in penumbra tissue and measured the levels of multiple inflammatory cytokines in the peripheral blood and brain. Furthermore, we used the HIF inhibitor, acriflavine hydrochloride (ACF), to determine the role of HIF in modulating the protective effects of RIPC against stroke. Finally, we studied the activation of the Akt and ERK signaling pathways in this process.

## Materials and Methods

All procedures in this study were conducted according to ethical standards and in accordance with the Declaration of Helsinki and with national and international guidelines and have been approved by the authors’ institutional review board. Male Sprague–Dawley (SD) rats, age-matched 19–20 months, were purchased from Vital River Laboratory Animal Technology Company Limited (Beijing, China). They were housed in a controlled room at a temperature of 24°C with a standard 12-h light/dark cycle, had free access to food and water and were randomly distributed into different groups. The number of animals in each group was 6–8.

### Middle Cerebral Artery Occlusion (MCAo)

Most pre-clinical stroke studies exclusively examined the response to stroke in young male animals. This is in part due to the difficulty of performing the surgeries, the high costs, and the poor survival rates in aged animals. In our experiment, rats were anesthetized with 3–5% isoflurane in 70% nitrous oxide and 30% oxygen and then maintained with 1–3% isoflurane. For the MCAo model, ischemia and reperfusion were established as previously described (Ren et al., 2011; Yang et al., 2018). As the aged rat weight was higher than that of younger ones, we chose a silica gel-coated nylon suture that was appropriate for rats up 450 g. The suture was withdrawn after 90 min of occlusion to allow for the re-opening of the MCA. Rat rectal temperature was maintained at 37 ± 0.5°C during the entire procedure. The cerebral blood flow (CBF) during the surgery before and after occlusion were measured by laser Doppler perfusion monitoring with a laser Doppler probe (PeriFlux System 5000, Perimed AB, Sweden) interfaced to a laptop equipped with the PeriSoft data acquisition software (PeriSoft Systems, Inc., Sweden) as previously described (Chen et al., 2018). The blood gas (PaCO_2_, PaO_2_ [mmHg] and pH) and blood sugar (mmol/L) were also examined during the surgery. The rats in the sham group were subjected to the surgical procedure without MCA occlusion.

### Remote Ischemic Preconditioning (RIPC)

RIPC was established as previously described (Yang et al., 2018). Briefly, 24 h before MCAo, RIPC was conducted on both hind limbs of rats anesthetized with 1–3% isoflurane by non-invasive occlusion of hind limb blood flow with a gauge bandage, in contrast to an invasive, direct femoral artery occlusion. The two hind limbs were simultaneously tied with a bandage to occlude blood circulation for 10 min and then released for 10 min to allow for reperfusion. The occlusion/reperfusion cycle was repeated three times.

### Behavioral Testing

Behavioral tests were conducted as described previously (Zhao et al., 2005; Yang et al., 2018). The behavioral tests were conducted to evaluate neurological functions after stroke in aged rats, including the tail hang test, home cage, and postural reflex. All behavioral tests were performed by a person who was blinded to the experimental conditions. We handled the rats for 3 days before surgery and tested their baseline on the day before surgery. All behavioral tests were then conducted 48 h after stroke onset.

For the tail hang test, the rat was hung by its tail approximately 10 cm above the floor. Stroke rats will turn to the contralateral side (left) of the ischemic hemisphere, and a head-turn of more than 90° was counted. Each time the rat was hung for no more than 5 s and each rat was hung for a total of 20 times. The percentage of head turns was calculated.

For the home cage limb test, the rat uses its forelimbs to explore the cage edge, and we counted the number of the times the ipsilateral, contralateral or both forelimbs touched the cage wall. A total of 20 contacts were recorded. The percentage of times that the ipsilateral forelimb was used was calculated using the formula: [ipsilateral + (both/2)] × 100%.

In the postural reflex test, the rat was placed on a table, its tail was held by one hand and the other hand was used to push its shoulder by nearly 20 cm three times. Non-ischemic rats gripped the table vigorously during the push, which was given a 0 score. A rat that showed less resistance and become stiff during the push was given a 1 score. A score of 2 was given if the rat had no resistance.

We also measured neurological deficits to evaluate stroke outcomes at 48 h after reperfusion using the method of the Longa scoring system, in which the scores were based on the following criteria: 0 = no deficit, 1 = failure to extend the left forepaw, 2 = circling to the left, 3 = falling to the left, 4 = failure to walk spontaneously and loss of consciousness, 5 = death.

### Drug Injections

To study the role of HIF in RIPC, a HIF inhibitor was used. Acriflavine hydrochloride (ACF, A8251, Sigma) was dissolved in phosphate-buffered saline (PBS), adjusted to a concentration of 2 mmol/L with additional PBS, and stored at 4°C. The ACF solution was given by intraperitoneal injection at a dose of 1.5 mg/kg, 24 h before stroke onset and every 24 h thereafter until euthanized (Ogle et al., 2012; Yang et al., 2018).

### Infarct Size Measurement

2,3,5-triphenyl-2H-tetrazolium chloride (TTC) staining was used for measuring the infarct size 48 h after reperfusion when the animals were euthanized. The non-ischemic hemisphere and the non-ischemic region in the ischemic hemisphere were measured, from which the infarct size was calculated according to the following formula: [(area of the non-ischemic hemisphere—area of the non-ischemic region in the ischemic hemisphere)/area of the non-ischemic hemisphere] × 100%. The detailed protocol has been described previously (Ren et al., 2009; Yang et al., 2018).

### Quantitative RT-PCR Analysis

To measure HIF-1α and HIF-2α mRNA expression, total RNA was isolated from the cortical penumbra, which was collected on ice and stored at −80°C immediately after the animals were euthanized. RNA was extracted with TRIzol reagent (Cat# 15596-026, Life Technologies, Carlsbad, CA, USA) according to the manufacturer’s instructions. The purified RNA was then reverse-transcribed into cDNA using the Reverse Transcription System (Cat# E6300S, New England BioLabs^®^ Inc., Ipswich, MA, USA). Quantitative RT-PCR analysis of the mRNA level of HIF-1α (HIF-1α F, GATGAATCAAAAGCAGTGACGAAGG, HIF-1α R, ATGCCTTAGCAGTGGTCATTTCTTG; HIF-2α F, ATCTTCCAGCCGCTCACC, HIF-2α R, GCCCACAGACCACTTCGTA) was performed using the SYBR Green Prime Script kit (RR420A, TAKARA). GAPDH (GAPDH F, TTCCTACCCCCAATGTATCCG; GAPDH R, CCACCCTGTTGCTGTAGCCATA) was chosen as the housekeeping gene. The real-time PCR program steps were: 95°C for 5 min, 45 cycles at 95°C for 5 s, 60°C for 5 s, and 72°C for 10 s, followed by 72°C for 1 min.

### Western Blotting

The cortical penumbra was collected on ice and stored at −80°C immediately after the animals were euthanized. Protein extractions were prepared with lysis buffer (50 mM Tris-HCl, pH 7.4, 150 mM NaCl, 5 mM EDTA, 0.5% NP-40, 0.1% Triton X-100, 0.1% SDS, 1 mM PMSF, 1× protein inhibitor mix Complete Mini). Then, 20 mg of protein per lane were separated by 10% polyacrylamide gel electrophoresis and transferred to nitrocellulose membranes for 2 h. The membranes were blocked with 5% skimmed milk for 1 h and then incubated overnight at 4°C with the primary antibody. Membranes were washed with TBST and incubated with the secondary antibody for 1 h. The signal was visualized by enhanced chemiluminescence. The primary antibodies used were anti-GAPDH (1:1000; Cat# 5174, Cell Signaling Technology, Inc., Danvers, MA, USA), anti-HIF-1α (1:1000; Cat# 14179, Cell Signaling Technology, Inc., Danvers, MA, USA), anti-HIF-2α (1:500; ab199, Abcam, Cambridge, MA, USA), anti-Akt (1:1,000, Cat# 4691, Cell Signaling Technology, Inc., Danvers, MA, USA), anti-p-Akt (1:1,000, Ser473, Cat# 4060, Cell Signaling Technology, Inc., Danvers, MA, USA), anti-ERK (1:1,000, Cat# 4695, Cell Signaling Technology, Inc., Danvers, MA, USA), and anti-p-ERK (1:1,000, Cat# 4370, Cell Signaling Technology, Inc., Danvers, MA, USA).

### AimPlex Multiplex Immunoassays

Peripheral blood and penumbra tissue were collected from the heart and the cortex of the ischemic brain, and an AimPlex rat inflammation assay kit (Beijing Quantobio, China) was used to detect multiple inflammatory cytokines in these samples. Six cytokines: IL-1β, IL-6, TNFα, IFN-γ, IL-4 and IL-10 were measured. The procedure was conducted according to the manufacturer’s instructions and as discussed previously (Yang et al., 2018). Briefly, 45 μl of capture bead working suspension and a 45 μl sample were sequentially shaken 750 times per minute for 60 min in the dark, followed by three washings in 100 μl washing buffer. The biotin-conjugated antibodies (25 μl) were added and the wells were sealed and shaken for 30 min in the dark. After washing three times with washing buffer, streptavidin-PE was added and they were shaken for 20 min in the dark. Finally, 100–200 μl 1 × Reading buffer was added to each well and the fluorescence signals of the samples were acquired using a flow cytometer (NovoCyte, ACEA Biosciences, Inc., San Diego, CA, USA), and the levels of the inflammatory factors were analyzed with FCAP Array 3.0 software.

### Statistical Analysis

Statistical analysis was performed using Prism 6 (GraphPad Software, Inc., La Jolla, CA, USA). Values are presented as means ± SEM. Differences between experimental groups were analyzed by Student’s *t*-test or one-way ANOVA with Newman-Keuls Multiple Comparison test as a *post hoc* test. A value of *P* < 0.05 was considered statistically significant. Before statistical analysis, data were checked for the Gaussian distribution and fitted the normal distribution.

## Results

### RIPC Protected Against Stroke in Aging Rats

**Figure 1:**
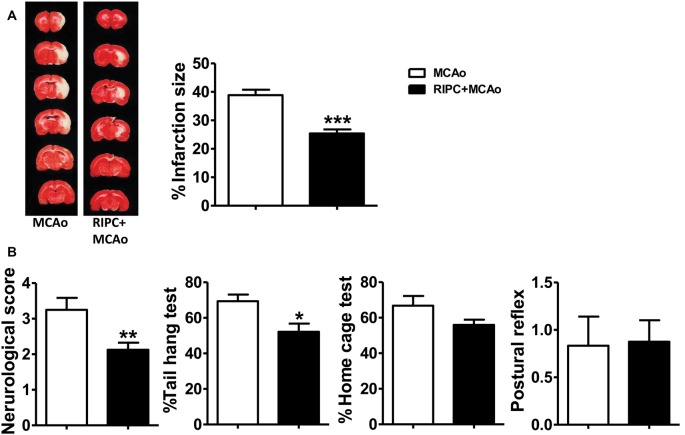
Remote ischemic preconditioning (RIPC) reduced infarct size and improved neurological function and the behavioral test after stroke in aged rats. **(A)** Representative images and infarct volume of 2,3,5-triphenyl-2H-tetrazolium chloride (TTC) staining in the MCAo and RIPC+MCAo group in aged rats. **(B)** Neurological score and behavior tests. Statistical analysis was performed by ANOVA. *,**,*** vs. MCAo, *p* < 0.05, 0.01, 0.001, respectively (*N* = 8–10 per group). MCAo, middle cerebral artery occlusion; RIPC, remote ischemic preconditioning.

In the previous study, we confirmed that RIPC was neuroprotective in young rats. In the current study, we used the same method we have used previously to detect the neuroprotective ability of RIPC in aged rats. The TTC staining results showed that RIPC induced a 34.64% significantly reduction of infarct size from 38.85 ± 1.15% to 25.39 ± 1.12% compared with MCAo (Figure 1A and Supplementary Figure S2), attenuated neurological scores (Figure 1B), and improved behavioral performance in the tail hang test (Figure 1B). Although there was no significant difference in the home cage test, RIPC showed a trend towards the promotion of functional recovery in aged rats. There was no significant difference in the postural reflex test (Figure 1B). Meanwhile, CBF detected during MCAo surgery had no change between MCAo and RIPC+MCAo group. It was reduced by nearly 80% of baseline during ischemia and reestablished to 78% of baseline following reperfusion (Supplementary Figure S1). There were no differences in blood gas and blood sugar between groups, either (Supplementary Tables S1, S2). These findings confirm that RIPC triggers structural and functional protection against stroke in aged rats.

### RIPC Decreased the Expression of HIF in the Ischemic Brain at 48 h Post-stroke

**Figure 2:**
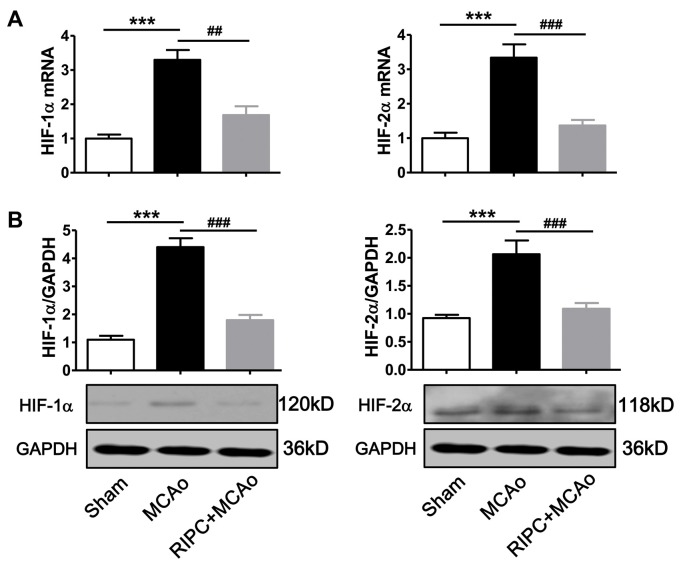
The expression of HIF-1α and HIF-2α in penumbra tissues. **(A)** The mRNA level of HIF-1α and HIF-2α was assessed by real-time RT-PCR. **(B)** Statistical data and representative images of western blot of HIF-1α and HIF-2α protein levels in penumbra tissues in the Sham, MCAo and RIPC+MCAo group. (*N* = 5–6 per group) Statistical analysis was performed by ANOVA. *** vs. Sham, *p* < 0.001, ^##,###^ vs. MCAo, *p* < 0.01, 0.001, respectively.

To test whether RIPC could regulate the expression of HIF-1α and HIF-2α in the ischemic tissue in aged rats, we measured HIF-1α and HIF-2α mRNA and protein levels in the penumbra tissue. The real-time RT-PCR results showed that mRNA expression of HIF-1α and HIF-2α were more than 3-fold upregulated 48 h post-stroke in the ischemic brain (Figure 2A), whereas RIPC decreased nearly 50% of HIF-1α and 60% of HIF-2α mRNA expression (Figure 2A). Similarly, the western blotting (WB) results (Figure 2B) and the statistical data showed that RIPC attenuated the protein expression of HIF-1α and HIF-2α in the ischemic brain 48 h post-stroke (Figure 2B).

### RIPC Downregulated Pro-inflammatory Factors in the Peripheral Blood and Brain

**Figure 3:**
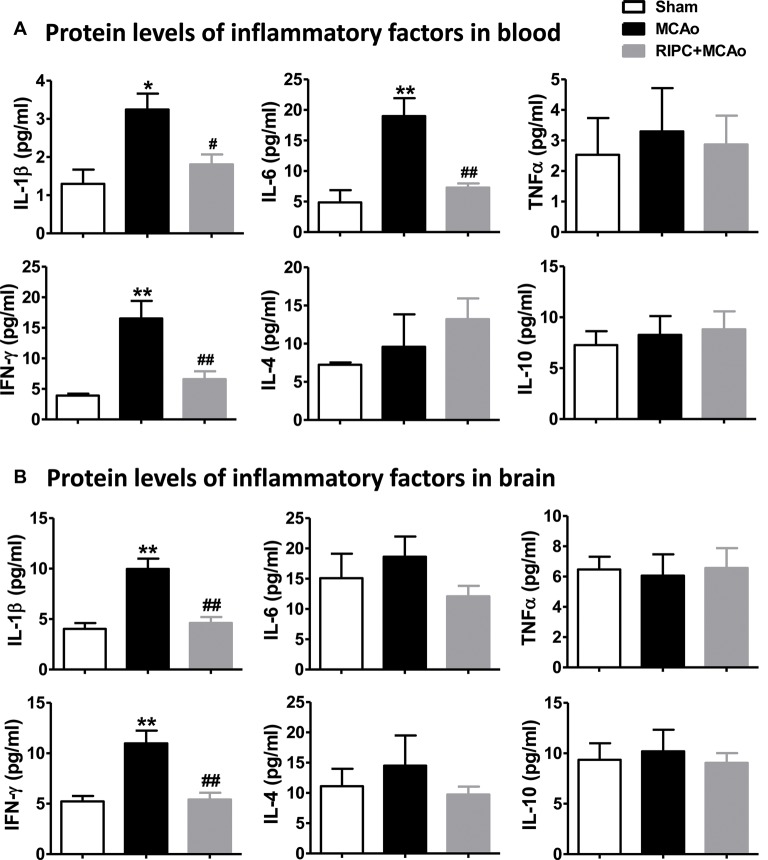
The effects of RIPC on inflammatory cytokines in the peripheral blood and brain. **(A)** Protein levels of the pro-inflammatory cytokines IL-1β, IL-6, TNFα and IFN-γ and anti-inflammatory cytokines IL-4 and IL-10, respectively, in the peripheral blood. A total of three groups were evaluated: Sham, MCAo, RIPC+MCAo. **(B)** Protein levels of IL-1β, IL-6, TNFα IFN-γ IL-4 and IL-10, in the ischemic brain. All samples were collected 48 h after stroke (*N* = 5–6 per group). *,** vs. Sham, *p* < 0.05, 0.01, ^#,##^ vs. MCAo, *p* < 0.05, 0.01, respectively.

To test if the inflammatory status were regulated by RIPC in aged rats after MCAo, we measured the effect of RIPC on the protein levels of the anti-inflammatory cytokines, IL-4 and IL-10, and that of the pro-inflammatory cytokines, IL-1β, IL-6, TNFα and IFN-γ in the peripheral blood and brain tissue. The results showed that MCAo increased the levels of pro-inflammatory cytokines while RIPC significantly decreased the levels of pro-inflammatory IL-1β, IL-6 and IFN-γ in the peripheral blood (Figure 3A) and that of IL-1β and IFN-γ in penumbra tissue (Figure 3B) 48 h post-stroke in aged rats. Taken together, these results suggest that RIPC reverses the increase in HIF expression caused by stroke and improves the level of inflammation in the ischemic and peripheral areas.

### HIF Inhibition Abolished the Protection of RIPC

**Figure 4:**
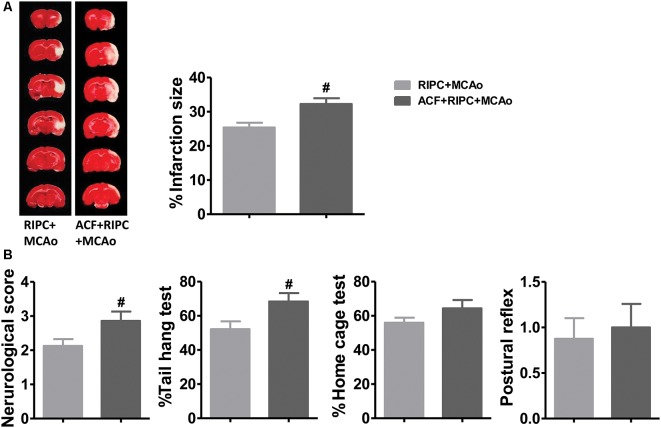
The effects of hypoxia-inducible factor (HIF) antagonist ACF on stroke outcomes in aged stroke animals treated with RIPC. **(A)** Representative images of TTC staining and the statistical results of infarct volume in the RIPC+MCAo and ACF+RIPC+MCAo group in aged rats. **(B)** Neurological score and behavioral tests of the tail hang test, home cage test, and postural reflex test (*N* = 8–10 per group). ^#^*P* < 0.05, vs. RIPC+MCAo. ACF, acriflavine hydrochloride, HIF inhibitor.

If HIF was necessary for the brain protection of RIPC in aged rats, inhibiting HIF activity would contribute to brain injuries induced by stroke. TTC staining results showed that the HIF inhibitor, ACF, abolished the protection of RIPC which ACF induced a 27.02% increase of the infarct size from 25.39 ± 1.12% to 32.25 ± 1.65% compared with RIPC (Figure 4A and Supplementary Figure S2). In addition, ACF also abolished the protective effects of RIPC as measured by neurological scores and behavioral performance including the tail hang test (Figure 4B). The initial decrease in CBF during ischemia and the restoration of CBF during reperfusion, the changes between groups were similar. Blood gas and blood sugar values had no differences between groups (Supplementary Figure S1 and Supplementary Tables S1, S2).

### HIF Inhibition Neutralized the Downregulation of Inflammatory Cytokines Induced by RIPC

**Figure 5:**
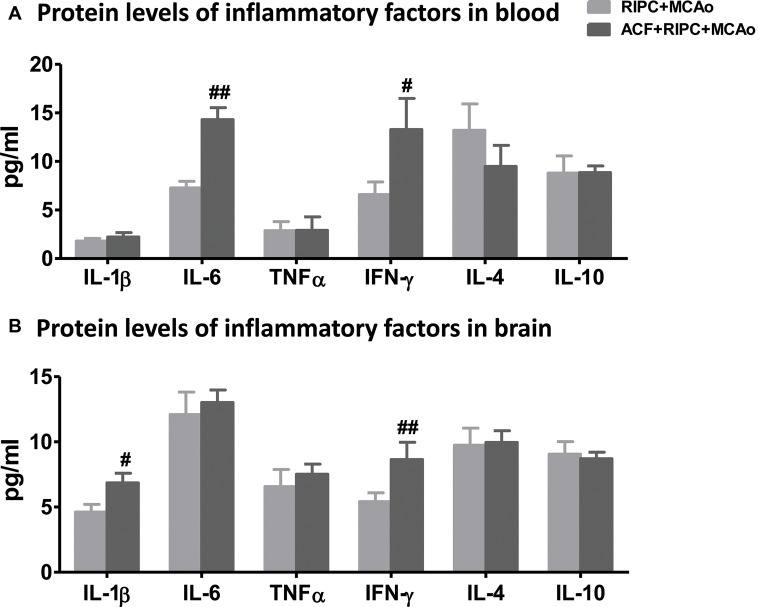
The effect of ACF on protein levels of inflammatory cytokines in aged stroke animals receiving RIPC. **(A)** Protein levels of the pro-inflammatory cytokines IL-1β, IL-6, TNFα and IFN-γ and anti-inflammatory cytokines IL-4 and IL-10 in the peripheral blood, respectively in RIPC+MCAo and ACF+RIPC+MCAo group in aged rats. **(B)** Protein levels of IL-1β, IL-6, TNFα, IFN-γ, IL-4 and IL-10 in the ischemic brain, respectively (*N* = 5–6 per group). ^#,##^, vs. RIPC+MCAo, *p* < 0.05, 0.01, respectively.

After examining the antagonistic effect of the HIF inhibitor with RIPC on brain injury, we further assessed its effect on inflammatory factors in aged rats. The results of the AimPlex multiplex immunoassays showed that in the peripheral blood, the HIF inhibitor ACF had an antagonist effect on IL-6 and IFN-γ (Figure 5A). ACF+RIPC+MCAo increased the expression of IL-6 and IFN-γ at the protein level compared with the RIPC+MCAo group. In addition, ACF had an antagonist effect on IL-1β and IFN-γ in the brain (Figure 5B). These results suggest that inhibition of HIF abolished the structural and functional protection of RIPC and neutralized the downregulation of inflammatory responses.

### RIPC Activated the Akt Signaling Pathway, While ACF Abolished It

**Figure 6:**
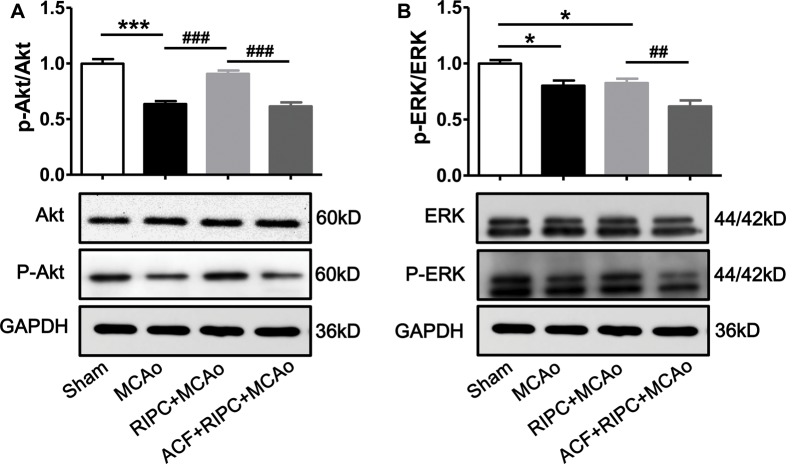
The effect of RIPC and ACF on the activation of Akt and ERK signaling pathway. **(A)** Statistical data of the ratio of p-Akt to total Akt and representative images of western blot of total Akt and p-Akt. A total of 4 groups were assessed: Sham, MCAo, RIPC+MCAo, ACF+RIPC+MCAo. **(B)** Statistical data of the ratio of p-ERK to total ERK and representative images of western blot of total ERK and p-ERK in penumbra tissues (*N* = 5–6 per group). Statistical analysis was performed by ANOVA. *,*** vs. Sham, *p* < 0.05, 0.001 respectively, ^##,###^ vs. MCAo, RIPC+MCAo, *p* < 0.01, 0.001, respectively.

Several studies have demonstrated that under ischemic conditions, the activation of the PI3k/Akt or EKR signaling pathway can be regulated by the expression of HIF, and signaling pathway activation can regulate inflammatory responses. We, therefore, investigated whether the Akt or ERK signaling pathway was involved in the action of RIPC to inhibit cerebral inflammation. Penumbra tissue was collected from the cortex of the ischemic brain. The results showed that the ratios of p-Akt/Akt (Figure 6A) and p-ERK/ERK (Figure 6B) were decreased 48 h post-stroke, whereas RIPC upregulated the expression of p-Akt not p-ERK (Figure 6). RIPC had no effect on the total expression of Akt and ERK. Moreover, ACF decreased the ratios of both p-Akt/Akt and p-ERK/ERK (Figure 6). These collective findings suggest that RIPC reverses the reduced expression of p-Akt following a stroke, while inhibition of HIF abolishes the reversion.

## Discussion

In the current study, first, we demonstrated that RIPC reduced the infarction size and improved neurological function in aged rats. Second, RIPC has been shown to decrease the expression of HIF-1α and HIF-2α in the ischemic brain and to decrease the protein levels of pro-inflammatory cytokines IL-1β and IFN-γ in both the blood and brain 48 h post-stroke. Third, the HIF-1α inhibitor, ACF, abolished the protective effects of RIPC on infarction and neurological function. Additionally, ACF inhibited the downregulation of pro-inflammatory factors induced by RIPC, including IL-6 and IFN-γ in blood and IL-1β and IFN-γ in the brain. Fourth, RIPC increased the ratio of p-Akt to total Akt, while ACF abolished the activation of the Akt signaling pathway in the brain. Collectively, these data showed that HIF-1α and HIF-2α played key roles in the protective effect of RIPC by modulating inflammatory responses likely mediated by the Akt signaling pathway in the brain and peripheral blood circulation in aged rats.

The success of most stroke models has not been well transformed into clinical practice. One potential reason for this failure is that stroke predominantly afflicts the elderly. Aging is a very important factor in stroke, and it has been found that there are many differences in stroke models between young and old animals such as infarction size, behavior, and expression and distribution of immune cells (Kuzumaki et al., 2010; Manwani et al., 2013; Orre et al., 2014; Zhao W. B. et al., 2017). In the previous study, we demonstrated that HIF-1α played a critical role in the protective effect of RIPC against stroke, which was likely achieved by modulating neuroinflammation in young rats (Yang et al., 2018). However, whether HIF plays a key role in RIPC in aged stroke is not addressed. Based on these, we chose an aged rats MCAo model and studied the protection and mechanism of RIPC post-stroke. We verified the infarction size was smaller in aged rats but there was worse neurological function than young rats. Further, our data suggested that RIPC protected against stroke not only in young rats but also in aged rats. RIPC reduced the infarction size and improved neurological function and behavior (Figure 1).

The HIF protein is a transcriptional factor that exists just a few minutes under normal oxygen levels but accumulates in hypoxic and ischemic cells (Ogle et al., 2012). Previous studies have shown that the neuroprotection against focal cerebral ischemia provided by various agents is mediated through the regulation of HIF (Doeppner et al., 2012; Wang et al., 2012; Meller and Simon, 2015). In the current study, we showed that MCAo upregulated the expression of HIF-1α and HIF-2α mRNA and protein levels in the ischemic brain of aged rats (Figure 2), which the evidence has been described in young animals in other reports (Zhong et al., 2012; Zhao et al., 2015; Zong et al., 2015). Further, we found that RIPC reversed this MCAo-induced upregulation of HIFs (Figure 2). It was suggested that RIPC improved the regional ischemia in the brain 48 h post-stroke in aged rats. Previous evidence showed that RIPC could regulate the expression of HIF, such as HIF-1α and HIF-2α, and played key roles in ischemic tissue including in the brain and heart in young experimental animals (Cai et al., 2008, 2013; Kapitsinou et al., 2014; Yang et al., 2018). To examine whether HIF also plays an important role in the neuroprotection of RIPC in aged rats, we used the heterodimer inhibitor, ACF, to inhibit HIF activity. The data showed that the injection of ACF abolished the structural and functional protection of RIPC on infarction size, neurological function and the tail hang test, one of the behavioral tests (Figure 4). These results demonstrated that HIF-1α and HIF-2α are key factors in the protection of RIPC in aged rats.

HIF is an inflammation mediator and can be stabilized by TNFα and IL-1β (Sharp and Bernaudin, 2004). It has been shown that IL-10 is the main target of HIF-1α in cardiomyocytes of circulating hypoxia-reoxygenation mice. RIPC can increase the plasma concentration of IL-10 and promote the subsequent remote activation of Akt signals in the heart (Cai et al., 2013). These findings are based on young animal models, and the changes in aged animals remain unclear. Our data showed that RIPC reduced the levels of pro-inflammatory cytokines IL-1β, IL-6 and IFN-γ in the blood, and IL-1β and IFN-γ in the brain in aged rats 48 h post-stroke (Figure 3), while the changes that were neutralized by the HIF inhibitor (Figure 5).

Several reports have identified under ischemic conditions, the activation of the Akt or EKR signaling pathway can be regulated by the expression of HIF (Hausenloy and Yellon, 2007; Cai et al., 2013; Martin-Puig et al., 2015; Meller and Simon, 2015; Ong et al., 2015). However, if HIF is directly affected by RIPC and further regulate the activity of Akt or EKR signaling pathway is unknown. Interestingly, in our study, we found that RIPC activated the Akt but not the ERK signaling pathway and that inhibition of HIF abolished the activation of Akt in aged rats following stroke (Figure 6). It suggested that HIF played key roles in RIPC reversed the reduced expression of p-Akt.

In the current study, there are several limitations. First, inhibitors of inflammatory cytokines and signaling pathways were not used. Thus, future studies should examine the infarct size when the inhibitors of inflammatory cytokines and signaling pathways are used; and come to a convincing conclusion the inflammatory responses and Akt/ERK signaling pathway is the cause of the reduced infarct size. Second, RIPC is an endogenous protective mechanism through which short-term sub-lethal ischemia in a remote organ can protect the major organ from further severe ischemia. However, due to the unpredictability of cerebral infarction, its clinical application is limited. Unlike ischemic preconditioning, ischemic postconditioning is a relatively novel concept, which follows reperfusion of a vessel and effectively inhibits inflammatory responses and delays cell death. So many researchers believe that ischemic postconditioning is more suitable for clinical transformation. However, to date, the evidence of cerebral ischemia postconditioning is limited to preclinical studies. Therefore, future studies will address the mechanism of remote ischemic postconditioning in the protection against cerebral ischemia, stimulate the clinical transformation of remote ischemic postconditioning.

## Conclusion

Our study provided strong evidence that RIPC protected against stroke in aged rats by modulating the expression of HIF-1α and HIF-2α in ischemic tissue, not only affecting the activation of the Akt pathway but also reducing the expression of pro-inflammatory cytokines in the peripheral blood and brain.

## Data Availability Statement

All datasets generated for this study are included in the article/Supplementary Material.

## Ethics Statement

The animal study was reviewed and approved by Capital Medical University Animal Review Committee.

## Author Contributions

XG, HD, and HZ: study concept and design. XD and JY: acquisition of data and wrote the draft. CL: made the figure and statistical analysis. SW and CZ: made a model. All authors contributed to the critical revision of the manuscript and have approved the final version of this review article.

## Conflict of Interest

The authors declare that the research was conducted in the absence of any commercial or financial relationships that could be construed as a potential conflict of interest.

## Supplementary Material

FIGURE S1 Cerebral blood flow during the MCAo surgery. Cerebral blood flow was measured at four time-points, baseline, 1 and 5 min of ischemia and reperfusion in the MCAo, RIPC+MCAo and ACF+RIPC+MCAo groups. Data were normalized to baseline and expressed as percentages.

FIGURE S2 Images of TTC staining. All the panels and representative images of TTC staining were presented. **(A)** MCAo group. **(B)** RIPC+MCAo group. **(C)** ACF+RIPC+MCAo group.

TABLE S1 Arterial blood gas parameter before and immediately after MCAo.

TABLE S2 Blood sugar value after surgery in each group.
