# Supplementary figures and images for: Hypoxia-Inducible Factor 1α and 2α Have Beneficial Effects in Remote Ischemic Preconditioning Against Stroke by Modulating Inflammatory Responses in Aged Rats
Source: Front Aging Neurosci. 2020 Mar 10;12:54. doi: 10.3389/fnagi.2020.00054 (PMC7076079; doi:10.3389/fnagi.2020.00054)

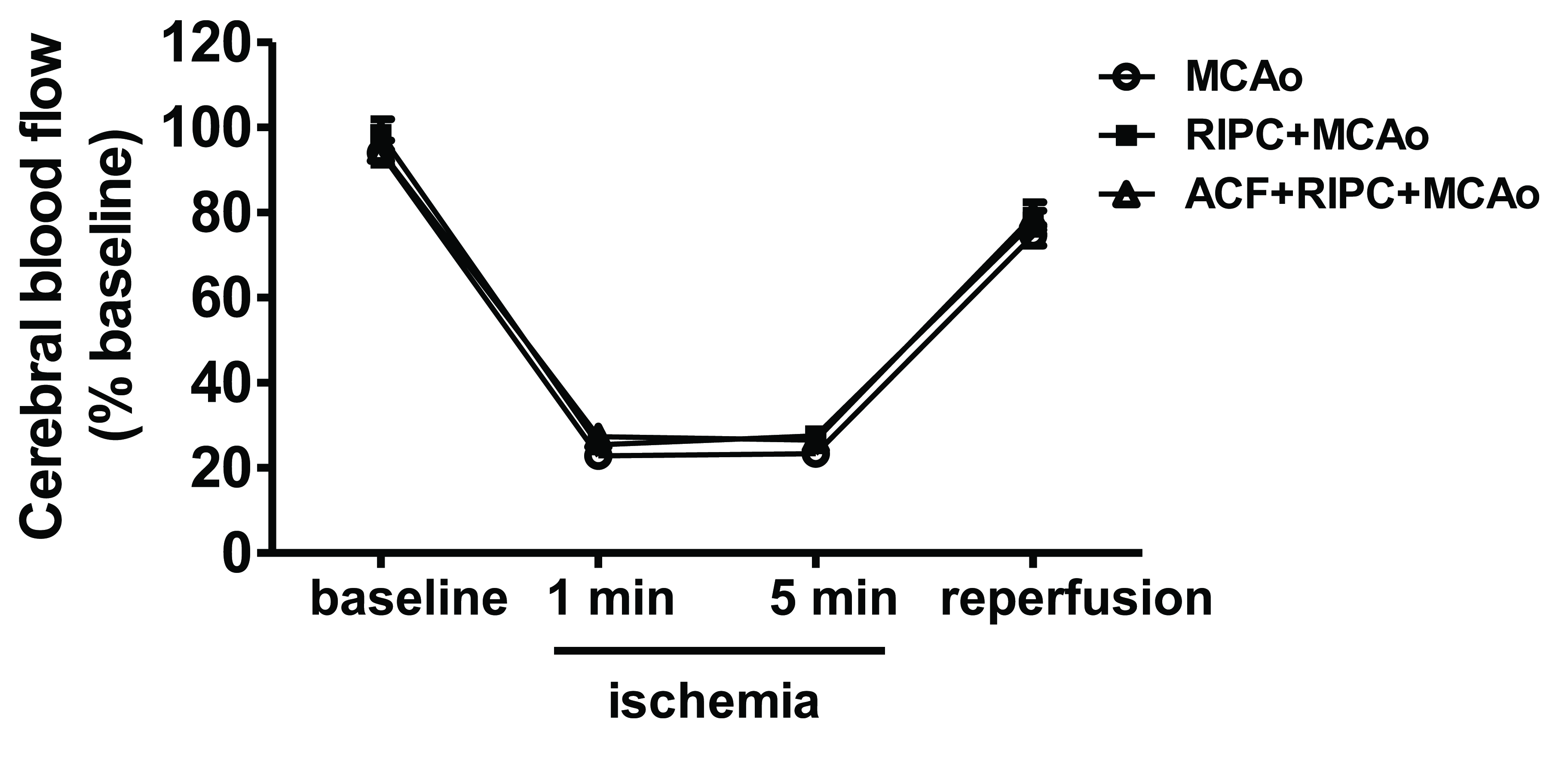

Supplement: FIGURE S1 — Cerebral blood flow during the MCAo surgery. Cerebral blood flow was measured at four time-points, baseline, 1 and 5 min of ischemia and reperfusion in the MCAo, RIPC+MCAo and ACF+RIPC+MCAo groups. Data were normalized to baseline and expressed as percentages. [file Image_1.TIF]

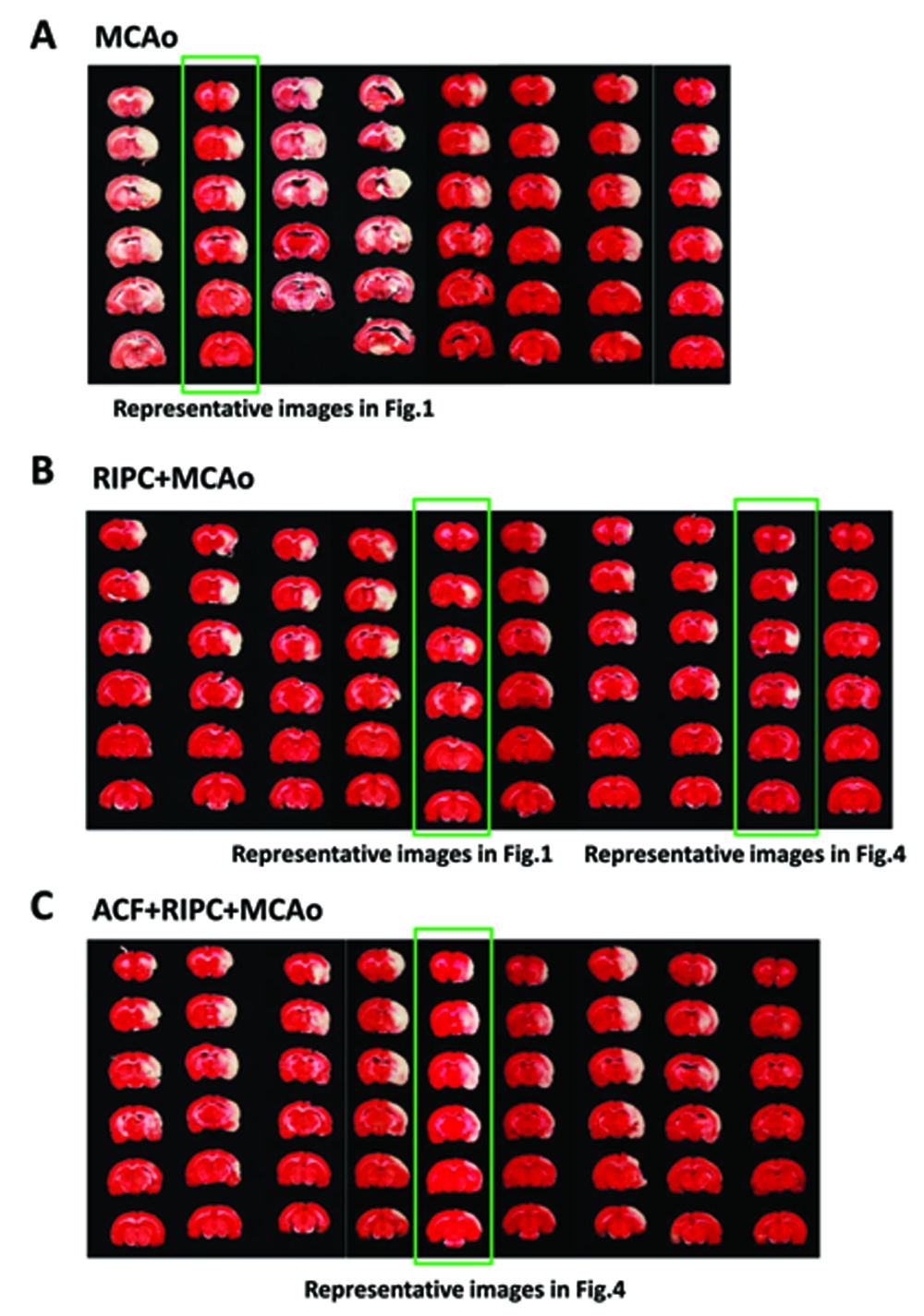

Supplement: FIGURE S2 — Images of TTC staining. All the panels and representative images of TTC staining were presented. (A) MCAo group. (B) RIPC+MCAo group. (C) ACF+RIPC+MCAo group. [file Image_2.TIF]

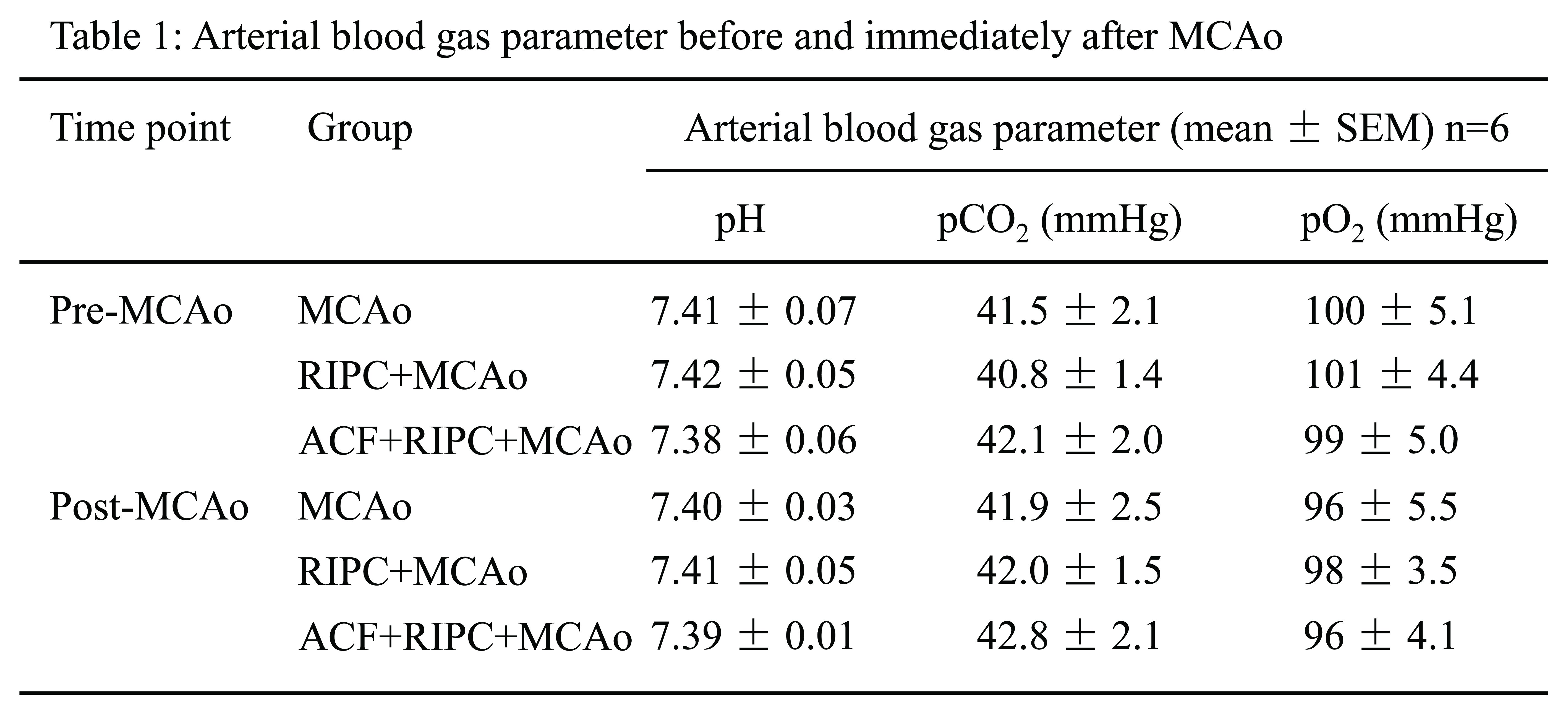

Supplement: TABLE S1 — Arterial blood gas parameter before and immediately after MCAo. [file Image_3.TIF]

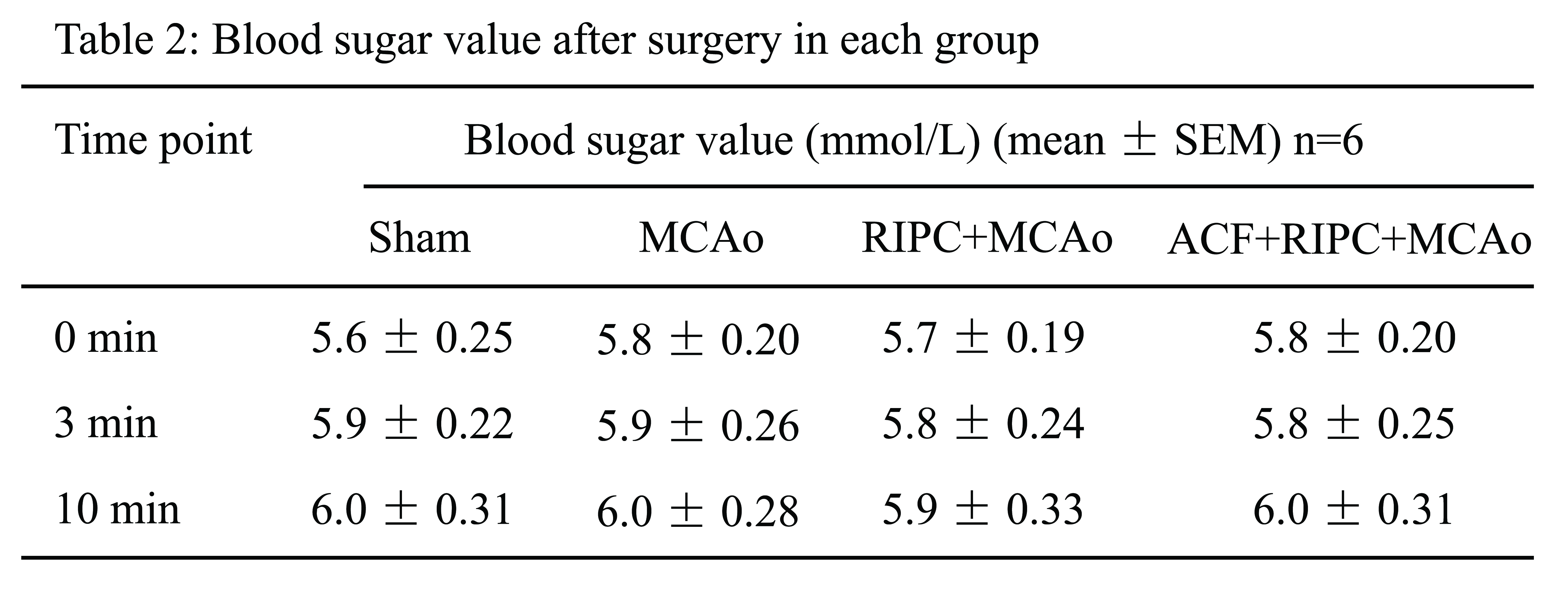

Supplement: TABLE S2 — Blood sugar value after surgery in each group. [file Image_4.TIF]
